# A novel survival algorithm in COVID-19 intensive care patients: the classification and regression tree (CRT) method

**DOI:** 10.4314/ahs.v21i3.16

**Published:** 2021-09

**Authors:** Sevinç Dağıstanlı, Süleyman Sönmez, Murat Ünsel, Emre Bozdağ, Ali Kocataş, Merve Boşat, Eray Yurtseven, Zeynep Çalışkan, Mehmet Güven Günver

**Affiliations:** 1 Kanuni Sultan Suleyman Research and Training Hospital, Department of General Surgery; 2 Kanuni Sultan Suleyman Research and Training Hospital, Department of Radiology; 3 Basaksehir Cam and Sakura City Hospital, Department of Anesthesiology and Reanimation; 4 Bezmialem Vakif University, Faculty of Health Sciences, Health Management; 5 Istanbul University, Istanbul Faculty of Medicine, Biostatistics; 6 Istanbul Yeni Yuzyil University, Medical Biochemistry

**Keywords:** Survival algorithm, COVID-19 intensive care patients, CRT method

## Abstract

**Background/aim:**

The present study aimed to create a decision tree for the identification of clinical, laboratory and radiological data of individuals with COVID-19 diagnosis or suspicion of Covid-19 in the Intensive Care Units of a Training and Research Hospital of the Ministry of Health on the European side of the city of Istanbul.

**Materials and Methods:**

The present study, which had a retrospective and sectional design, covered all the 97 patients treated with Covid-19 diagnosis or suspicion of COVID-19 in the intensive care unit between 12 March and 30 April 2020. In all cases who had symptoms admitted to the COVID-19 clinic, nasal swab samples were taken and thoracic CT was performed when considered necessary by the physician, radiological findings were interpreted, clinical and laboratory data were included to create the decision tree.

**Results:**

A total of 61 (21 women, 40 men) of the cases included in the study died, and 36 were discharged with a cure from the intensive care process. By using the decision tree algorithm created in this study, dead cases will be predicted at a rate of 95%, and those who survive will be predicted at a rate of 81%. The overall accuracy rate of the model was found at 90%.

**Conclusions:**

There were no differences in terms of gender between dead and live patients. Those who died were older, had lower MON, MPV, and had higher D-Dimer values than those who survived.

## Introduction

Corona Virus Disease 2019 (COVID-19) was first reported in the city of Wuhan in China in December 2019. Here, the newly identified human-coronavirus was named temporarily as 2019-nCoV, and then, with a common consensus, renamed as Severe Acute Respiratory Syndrome-Related Coronavirus “SARS-CoV-2” 1. SARS-CoV-2 spread quickly around the world from Wuhan, China, where it was first announced, and was declared as pandemia by the WHO on March 11, 2020. Again, on the same day, it was announced by the Ministry of Health that the first case was detected in Turkey, and on March 17, 2020, the first death occurred due to SARS-CoV-2. As of May 12, 2020, it was announced as current figures that the total number of cases was 141.475, there were 3894 total deaths, the number of patients in intensive care was 1045, and 576 people were intubated.

With the spread of SARS-CoV-2 among countries, thanks to the strong healthcare service structure of the Ministry of Health of the Republic of Turkey, and by seeing the processes experienced by other countries, necessary precautions, measures and emergency action plans were performed quickly. In this process, the kits that were required for PCR tests were brought to the country, all private, foundation, university and public hospitals that had 3rd Level Intensive Care Units, and at least two of the chest diseases, infectious diseases and internal expertise services, were converted into pandemia hospitals and provided free services for all citizens in the framework of SARS-CoV-2.

CT devices were quickly included in use for SARS-CoV-2 scans when PCR test is insufficient or incomplete, and were quickly reported by radiologists who were specialized in the field. In the light of the algorithms that were updated by the Ministry of Health and according to CT and clinical findings, it was ensured that patients were quarantined in services or in intensive care units or at homes to prevent rapid spread. Many people with COVID-19 infection face the disease mildly, and have flu-like symptoms like fever and cough, others may be asymptomatic, and some patients may have Acute Respiratory Distress Syndrome (ARDS). The patients who develop ARDS may require respiratory support, and for this reason, the need for intensive care may appear. Initial reports published show that approximately 5% of proven COVID-19 infections are associated with serious disease that require intensive care 2.

In light of these reports, according to 2018 data, with 38,908 intensive care beds capacity, considering the intensive care and intubation requirement that might increase, all surgeries -except for emergency surgeries were cancelled especially in Istanbul, the most populous city of the country with a population of 15 million 519 thousand 267 people, and surgery rooms and other related areas in many other cities have been converted into intensive care units. For this reason, the present study aimed to create a decision tree for the identification of clinical, laboratory and radiological data of individuals with COVID-19 diagnosis or suspicion of Covid-19 in the Intensive Care Units of a Training and Research Hospital of the Ministry of Health on the European side of the city of Istanbul.

## Material and Methods

The present study, which had retrospective and sectional design, covered all the patients treated with Covid-19 diagnosis or suspicion of Covid-19 in the intensive care of Kanuni Sultan Suleyman Research and Training Hospital between 12 March and 30 April, 2020. Firstly, the approval for the study was obtained from the Chief Physician; application for permission was made to the Ministry of Health, and the approval was obtained from the Ethics Committee of Science, Social and Non-Interventional Health Sciences Research at Istanbul Yeni Yuzyıl University as a result of the meeting on 11.05.2020 with the number 2020/04-05.

The number of the beds in the hospital where the study was conducted is 648. It also has a total of 40 intensive care beds with ventilator devices. In addition, there are 32 beds with ventilator device in the 3rd Stage and 8 beds ventilator device in the 2nd Stage for adults. There are a total of 4 CT devices in our hospital, and 3 CT Devices were gradually allocated only for Covid-19-suspicious cases, depending on patient density. No sampling method was used in the study, and a total of 97 cases were included. In all cases who had symptoms (fever, cough, shortness of breath, etc.) admitted to the Covid-19 clinic, nasal swab samples were taken and thoracic CT was performed when considered necessary by the physician, radiological findings were interpreted (atypical, typical-mild, typical-moderate-severe), and clinical and laboratory data were included to create the decision tree. The following were considered in the interpretation of the CT results. Atypical: Unilateral ground glass density or consolidated areas. Typical (mild): (Lung parenchyma involvement 1–25%): Bilateral peripheral-weighted patchy focal multiple ground glass density areas. Typical (moderate-severe) (Lung parenchyma involvement 25–100%): Bilateral, widespread and consolidated multiple ground glass densities in peripheral-weighted patchy form combining at places in the upper and middle lobes of both lungs.

The CTs were performed in Somatom Definition AS+, Siemens Healthineers, Germany CT Device. The patients were in supine position. 16-dedector 0.75mm section thickness tube voltage = 120 kVp, automatic tube current modulation (30 - 70 mAs), pitch = 0.99 - 1.22 mm, matrix = 512 × 512, slice thickness = 10 mm, field of view = 350 mm × 350 mm.

A decision tree was created in which the laboratory data and imaging results of the cases recorded during the hospital admission step, alanine aminotransferase (ALT), Albumin, aspartate aminotransferase (AST), C-reactive protein (CRP), Ferritin, basophils (BASO), eosinophils (EO), hematocrit (HTC), hemoglobin (HGB), lymphocytes (LYM), monocyte (MON), mean platelet volume (MPV), Neutrophils (NEU), platelet (PLT), red blood cell (RBC), white blood cell (WBC), creatine kinase (CK), lactate dehydrogenase (LDH), procalcitonin (Procal), troponin I (Trop_I), D_dimer laboratory data were taken as independent variables, and survival was taken as dependent variable. The Classification and Regression Tree (CRT) algorithm was applied to these variables 3. The result of the Computerized Tomography (CT) was used as the influence variable (2) in the decision tree 4.

## Results

A total of 97 cases who were diagnosed with Covid-19 with PCR testing or CT findings and who were referred to intensive care at Kanuni Sultan Suleyman Training and Research Hospital were evaluated in this study. A total of 61 of the cases included in the study died, and 36 were discharged with cure from the intensive care process (Table-1).

**Table 1:** Survival status of Covid-19 intensive care patients by gender

p=0.913		Gender		Total
		Female	Male	
Survival	Dead	21	40	61
	Live	12	24	36
Total		33	64	97

The baseline (before the intensive care) characteristics of the relevant cases are as follows.

As seen in Table 2;

- There were no differences in terms of gender between dead and live patients.
- Those who died were older than those who survived.
- Those who died had lower MON values than those who survived.
- Those who died had lower MPV values than those who survived.
- Those who died had higher D_Dimer value than those who survived.

**Table 2:** Baseline Characteristics of Covid-19 intensive care patients

Group Statistics						
Survival		N	Mean	Std. Deviation	Std. Error Mean	p
Age	Dead	61	68.38	11.60	1.49	0.0000
	Live	36	53.69	18.89	3.15	
ALT	Dead	61	34.57	24.50	3.14	0.3076
	Live	36	29.61	20.23	3.37	
Albumin	Dead	61	37.72	39.54	5.06	0.6519
	Live	36	34.72	5.48	0.91	
AST	Dead	61	51.05	35.84	4.59	0.2163
	Live	36	41.25	40.11	6.69	
CRP	Dead	61	138.48	102.53	13.13	0.3443
	Live	35	220.20	660.23	111.60	
Ferritin	Dead	58	871.36	893.98	117.39	0.4805
	Live	34	737.81	834.96	143.19	
BASO (10^9/L)	Dead	61	0.04	0.13	0.02	0.3133
	Live	36	0.02	0.02	0.00	
EO (10^9/ L)	Dead	60	0.02	0.07	0.01	0.5113
	Live	36	0.03	0.07	0.01	
Htc (%)	Dead	61	36.31	6.98	0.89	0.4272
	Live	36	35.12	7.31	1.22	
HGB (g/dL)	Dead	61	12.16	2.44	0.31	0.5742
	Live	36	11.87	2.55	0.43	
LYM (10^9/L)	Dead	61	1.33	2.45	0.31	0.7372
	Live	36	1.19	0.62	0.10	
MON (10^9/L)	Dead	61	0.48	0.28	0.04	0.0366
	Live	36	0.61	0.36	0.06	
MPV (fL)	Dead	60	10.27	1.22	0.16	0.0243
	Live	35	10.80	0.81	0.14	
NEU (10^9/L)	Dead	61	117.77	864.63	110.71	0.4439
	Live	36	6.75	3.86	0.64	
PLT (10^9/L)	Dead	61	206,295.08	100,749.09	12,899.60	0.1815
	Live	36	233,527.78	88,058.26	14,676.38	
RBC (10^9/L)	Dead	61	11.79	58.09	7.44	0.4373
	Live	36	4.22	0.91	0.15	
WBC	Dead	61	12,178.76	29,619.03	3,792.33	0.3892
	Live	36	7,871.96	4,418.98	736.50	
CK	Dead	47	416.66	924.77	134.89	0.2794
	Live	33	233.00	338.07	58.85	
LDH	Dead	47	436.98	241.53	35.23	0.2807
	Live	29	378.65	201.75	37.46	
Procal	Dead	59	13.92	87.36	11.37	0.4096
	Live	36	1.82	6.21	1.04	
Trop_I	Dead	49	0.07	0.13	0.02	0.3159
	Live	30	0.21	0.92	0.17	
D_ dimer	Dead	53	9.86	11.87	1.63	0.0082
	Live	33	3.74	6.68	1.16	

The Survival - CT Cross Table of the cases transferred to intensive care are given below (Table-3).

**Table 3:** Survival - CT comparison of the Covid-19 intensive care patients

p=0.406		CT_New Grouping			Total
		Typical (Mild)	Typical (Moderate- Severe)	Atypical	
Survival	Dead	6	42	13	61
	Live	7	22	7	36
Total		13	64	20	97

A Decision Tree was created in which baseline laboratory values, age, gender and laboratory data were included as the independent variables, and survival status as dependent variable, and the analyses were conducted by using the CT results as the influence variable applying the CRT algorithm. The influence variable is the intermediary variable, which is not included in the structure of the tree, but is used in calculations for the creation of such trees 3, 4. The predicted/observed matrix of the decision tree is below (Table 4).

**Table 4:** Decision tree success table of Covid-19 intensive care patients

Observed	Predicted		
	Dead	Survival	Accuracy Percentage
Dead	58	3	95.1%
Survival	7	29	80.6%
Overall Percentage	67.0%	33.0%	**89.7%**

**Figure 1:**
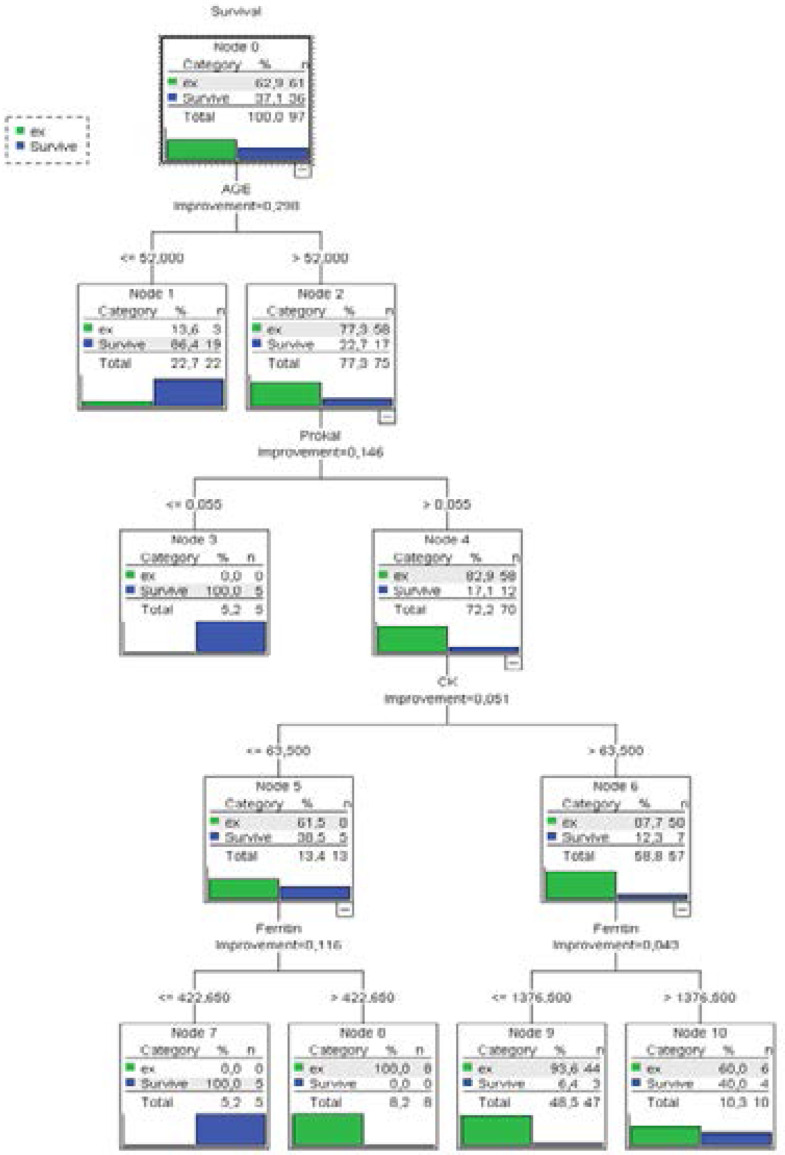
CRT Decision Tree

By using the de c ision tree algorithm created in this study (fig-1), dead cases will be predicted at a rate of 95%, and those who survive will be predicted at a rate of 81%. The overall accuracy rate of the model was found as 90%.

## Discussion

COVID-19 emerged before us as a threat for the preparations of all countries and biosecurity conditions. Clinical data, laboratory findings, and radiological imaging results provide clinicians with critical information, which must be examined carefully when a new infectious disease appears.

Among current diagnostic methods for COVID-19, there are detection of the virus with genomic techniques by using Polymerase Chain Reaction (PCR)-based methods or deep sequence method. The fact that viral genome is not adequate in the amplifiable sample collection area in these detection methods, viral replication does not have a time window, incorrect sample collection are among the reasons, which can limit the usability of the quantitative PCR (qPCR)-based analysis^5^–^7^. Such limitations can cause false negative diagnoses, and cause such patients spread the infection rapidly, prevent the efforts spent to avoid the spread of the virus, and produce serious consequences. Because of these reasons, laboratory tests and imaging methods are important in identifying fake PCR-negative patients. In the present study, the relation between mortality in intensive care patients and epidemiological, laboratory and imaging was investigated.

As mentioned in previous studies, it was found in our study that age has a big effect on mortality. As the age increases, so do mortality rates. The mortality rates increased when the age increased above 52 in the decision tree mentioned above. The procalcitonin value is of great importance in these patients, especially in significant laboratory findings of inflammation. In a study conducted retrospectively on 191 patients in China, it was shown that Covid-19-related in-hospital mortality rates increased with increased age 8.

In a multi-center study conducted on 150 patient records in China, significant differences were found in terms of age among the survivors and those who died^9^. It can be speculated that our results coincide with the results of the studies conducted on age.

Patients with procalcitonin values below 0.055 ng/ml who were included in the study had 100% survival rates. The mortality rate was measured to be 82.9% in people with procalcitonin level above 0.055 ng/ml. In a compilation study 10, it was emphasized that high serum procalcitonin and ferritin values showed poor prognostic factors.

In a previous study 11, the data obtained in terms of Covid-19 and laboratory parameters were published in the form of a letter to the editor, and it was reported that procalcitonin values were increased in patients admitted to the Intensive Care Unit. These two studies show parallel results with the present study of ours.

In patients who had procalcitonin values above 0.055 ng/ml, the CK value, which is one of the important enzymes in our body, was an important parameter; and mortality was measured to be 87.7% in patients with CK >63.5 compared to 61.5% in patients with CK <63.5. In a single-center study conducted on 138 hospitalized patients admitted to intensive care unit in China, higher levels of D-dimer and creatine kinase were found in laboratory findings 12. In the present study, similarly, it was found that the creatine kinase and D-dimer values were high in intensive care patients.

In the final step of the decision tree, the acute phase reactant ferritin values are important. The mortality rate was 0% in patients with <422.65 ferritin value and CK<63.5, it was 100% in patients with >422.65. In addition, while the mortality rate was 93.6% in patients with ferritin value <1376.5 and CK>63.5, it was 60% in patients with 1376.5 ferritin value. In a meta-analysis study 13, it was emphasized that serum ferritin levels were a powerful differential parameter in the severity of the disease. In our study, the ferritin levels appeared before us as a strong differential parameter in intensive care patients.

In a study conducted on 201 Covid-19 patients in China, it was shown that the high serum ferritin levels were associated with ARDS development; however, these levels did not tend to have a relation with survival. However, in our study, it was found that there was a relation between ferritin levels and survival in our patients 14.

In a previous study 8 conducted with 191 patients, similar to our study, higher serum ferritin levels and higher mortality rates were associated in the single-variable analysis, and they did not provide a multivariate analysis. In addition, according to baseline statistics, the D_Dimer values of dead individuals were higher than the surviving individuals. In a multi-center study conducted with 1099 Covid-19 patients, it was found that D-Dimer values increased in 46.4% of patients, and that these values was higher at significant levels in severe Covid-19 cases 15.

In a previous study, it was concluded that the D-dimer levels were higher in patients who needed intensive care support (Median D-dimer 2.4 mg / L for ICU, 0.5 mg / L for non-ICU) 16.

## Conclusion

In the present study, it was found that age is an important factor in patients treated with Covid-19 diagnosis or suspicion of it in intensive care. It was observed that the levels of procalcitonin, which is an indicator of inflammation in the decision tree, and acute phase reactant ferritin values were important mortality parameters. Although the limitation of the present study was the number of patients, studies conducted on Covid-19 patients who need intensive care are extremely limited. The biggest problem in pandemics is that intensive care units become unable to serve in this process despite the rate of spread of the disease. For this reason, the subject of the present study that was investigated will provide us information about the progress of the disease in intensive care and before, and the contribution of our study to the literature is undeniable. In this respect, it is recommended to conduct more comprehensive studies with larger groups for intensive care patients with the data obtained here.

## Figures and Tables

**Figure 2:**
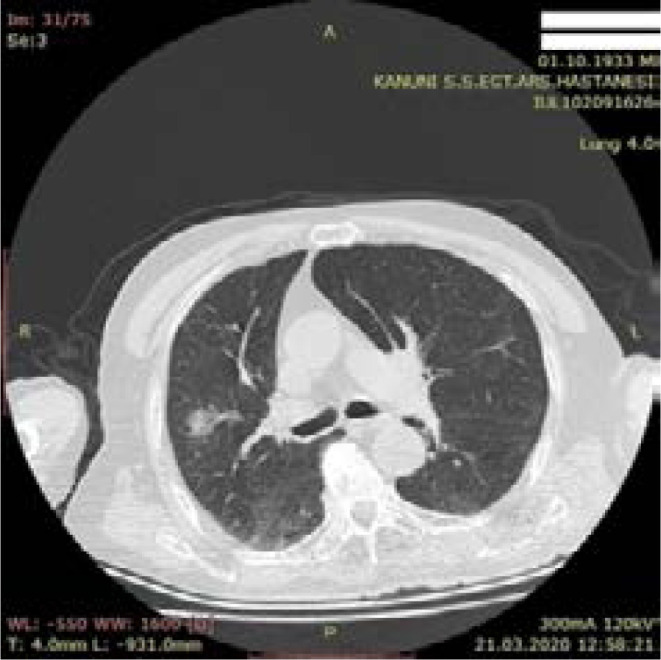
Atypical CT finding - 86-year-old male patient - Dead - Focal ground glass density in right lung upper lobe

**Figure 3:**
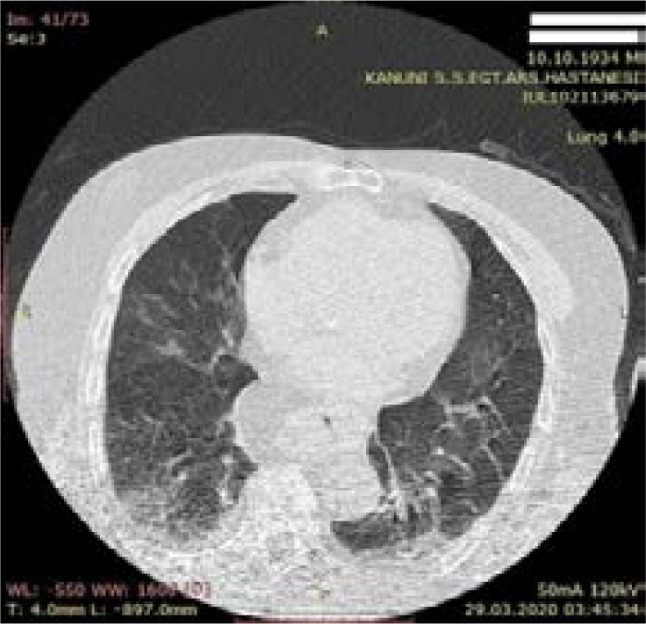
Typical mild CT finding - 85-year-old male patient - Dead - peripheral ground glass density in the lower lobe of both lungs

**Figure 4:**
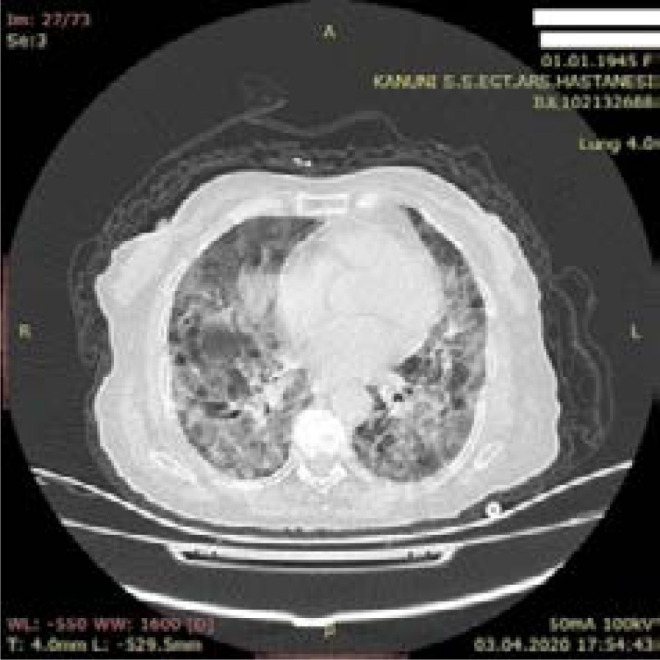
Typical - (Severe) CT finding - 75-year-old female patient - Dead - Multiple patchy ground glass densities combined at places in both lungs

**Figure 5:**
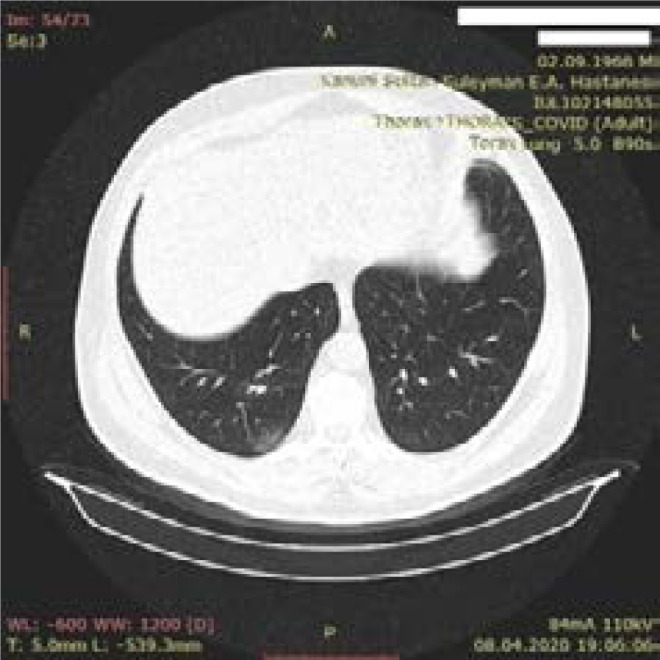
Atypical CT finding - 53-year-old male patient - peripheral focal ground glass density in right lung lower lobe

**Figure 6:**
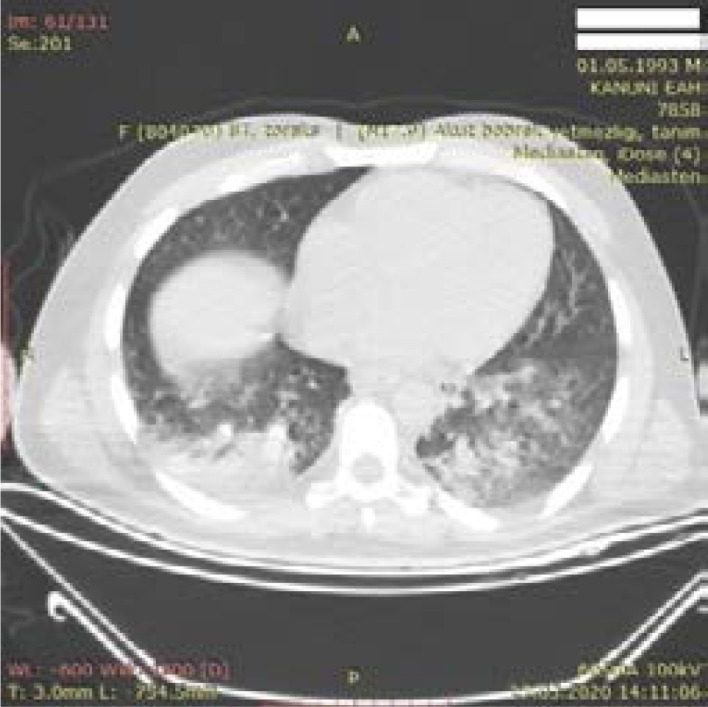
Typical medial CT finding - 27-year-old female patient – Ground glass densities in consolidated form in lower lobes of both lungs

**Figure 7:**
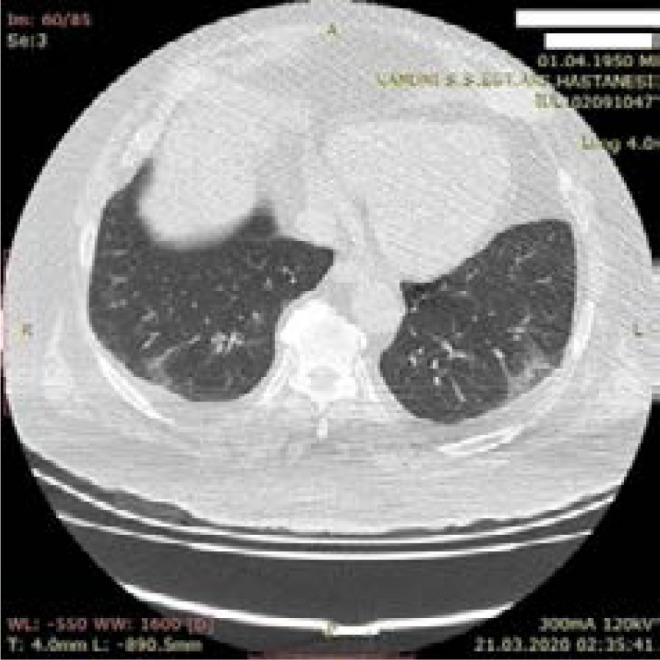
Typical mild CT finding - 60-year-old male patient -peripheral focal ground glass densities in lower lobes of both lungs
